# The diagnosis of pre-eclampsia using two revised classifications in the Finnish Pre-eclampsia Consortium (FINNPEC) cohort

**DOI:** 10.1186/s12884-016-1010-0

**Published:** 2016-08-12

**Authors:** Jenni Kallela, Tiina Jääskeläinen, Eija Kortelainen, Seppo Heinonen, Eero Kajantie, Juha Kere, Katja Kivinen, Anneli Pouta, Hannele Laivuori

**Affiliations:** 1 Medical and Clinical Genetics, University of Helsinki and Helsinki University Hospital, Helsinki, Finland; 2 Obstetrics and Gynaecology, University of Helsinki and Helsinki University Hospital, Helsinki, Finland; 3 Chronic Disease Prevention Unit, National Institute for Health and Welfare, Helsinki, Finland; 4 Children’s Hospital, Helsinki University Hospital and University of Helsinki, Helsinki, Finland; 5 PEDEGO Research Unit, MRC Oulu, Oulu University Hospital and University of Oulu, Oulu, Finland; 6 Department of Biosciences and Nutrition, and Science for Life Laboratory, Karolinska Institutet, Stockholm, Sweden; 7 Molecular Neurology Research Program, University of Helsinki, Helsinki, Finland; 8 Folkhälsan Institute of Genetics, Helsinki, Finland; 9 Division of Cardiovascular Medicine, University of Cambridge, Cambridge, UK; 10 Department of Government services, National Institute for Health and Welfare, Helsinki, Finland; 11 Institute for Molecular Medicine Finland, University of Helsinki, Helsinki, Finland

**Keywords:** Pre-eclampsia, Gestational hypertension, Proteinuria, Classification, Criteria

## Abstract

**Background:**

The Finnish Pre-eclampsia Consortium (FINNPEC) case-control cohort consisting of 1447 pre-eclamptic and 1068 non-pre-eclamptic women was recruited during 2008–2011 to study genetic background of pre-eclampsia and foetal growth. Pre-eclampsia was defined by hypertension and proteinuria according to the American College of Obstetricians and Gynecologists (ACOG) 2002 classification. The ACOG Task Force Report on Hypertension in Pregnancy (2013) and The International Society for the Study of Hypertension in Pregnancy (ISSHP) (2014) have published new classifications, in which proteinuria is not necessary for diagnosis when specific symptoms are present. For diagnoses based on proteinuria, the ISSHP 2014 criteria raised its threshold to 2+ on dipstick. We studied how the new classifications would affect pre-eclampsia diagnoses in the FINNPEC cohort.

**Methods:**

We re-evaluated pre-eclampsia diagnosis using the ACOG 2013 and the ISSHP 2014 classifications in pre-eclamptic women whose proteinuria did not exceed 1+ on dipstick (*n* = 68), in women with gestational hypertension (*n* = 138) and in women with chronic hypertension (*n* = 66).

**Results:**

The number of women with pre-eclampsia increased 0.8 % (1459/1447) according to the ACOG 2013 criteria and 0.6 % (1455/1447) according to the ISSHP 2014 criteria. All 68 women with the amount of proteinuria not exceeding 1+ on dipstick diagnosed originally pre-eclamptic met the ACOG 2013 criteria but only 20 women (29.4 %) met the ISSHP 2014 criteria. Seven (5.1 %) and 35 (25.4 %) women with gestational hypertension were diagnosed with pre-eclampsia according to the ACOG 2013 and the ISSHP 2014 criteria, respectively. Correspondingly five (7.6 %) and 21 (31.8 %) women with chronic hypertension were diagnosed with pre-eclampsia according to the ACOG 2 013 and the ISSHP 2014 criteria.

**Conclusions:**

Only minor changes were observed in the total number of pre-eclamptic women in the FINNPEC cohort when comparing the ACOC 2002 classification with the ACOG 2013 and ISSHP 2014 classifications.

## Background

Pre-eclampsia (PE) is a pregnancy-specific vascular disorder, the pathogenesis of which is still not completely understood. Symptoms appear usually late in the third trimester [1]. It is one of the leading causes of maternal and neonatal morbidity and mortality. PE is characterised by vascular endothelial dysfunction and placental implantation abnormalities, causing perfusion problems and in some cases intrauterine growth restriction [2]. PE resolves postpartum after the delivery of the placenta. Prediction and prevention have proven to be difficult due to the complex nature of the disease [3]. There are major implications for the long-term health of the mother and the newborn. PE is associated with elevated risk to develop cardiovascular diseases later in life [4–6]. Affecting approximately 3-5 % of pregnancies and causing as much as 10 % of pregnancy related complications, better diagnostic criteria are needed to improve the recognition of PE and its diverse subtypes.

Until the last few years, the core criteria of PE have been considered to be a new onset hypertension after the 20^th^ gestational week combined with proteinuria ≥ 300 mg per day. Heterogeneity of the disorder is more and more appreciated and therefore new diagnostic criteria have recently been introduced. Proteinuria has been questioned as a sine qua non [7]. According to the two new diagnostic criteria by The American College of Obstetricians and Gynecologists (ACOG) in 2013 [8] and International Society for the Study of Hypertension in Pregnancy (ISSHP) in 2014 [7], new onset hypertension in the absence of proteinuria but combined with haematological complications, renal insufficiency, impaired liver function, neurological symptoms, or uteroplacental dysfunction also fulfil diagnostic criteria for PE. This is to provide a more broad definition of PE for clinical practice leaving proteinuria to ensure specificity of the diagnosis in scientific purposes [7]. More sensitive recognition is beneficial considering the potential severity of the disorder. Currently PE is diagnosed based on clinical characteristics but biomarkers and genetic variants are expected to provide more specific criteria in the future.

We examined how the new criteria affected the PE diagnosis in the Finnish Genetics Pre-eclampsia Consortium (FINNPEC) cohort. Originally, PE was defined by hypertension and proteinuria according to the ACOG 2002 classification [9]. Three subgroups were re-evaluated; PE women with the amount of proteinuria not exceeding 1+ on dipstick, women with gestational hypertension, and women with chronic hypertension.

## Methods

**Fig. 1:**
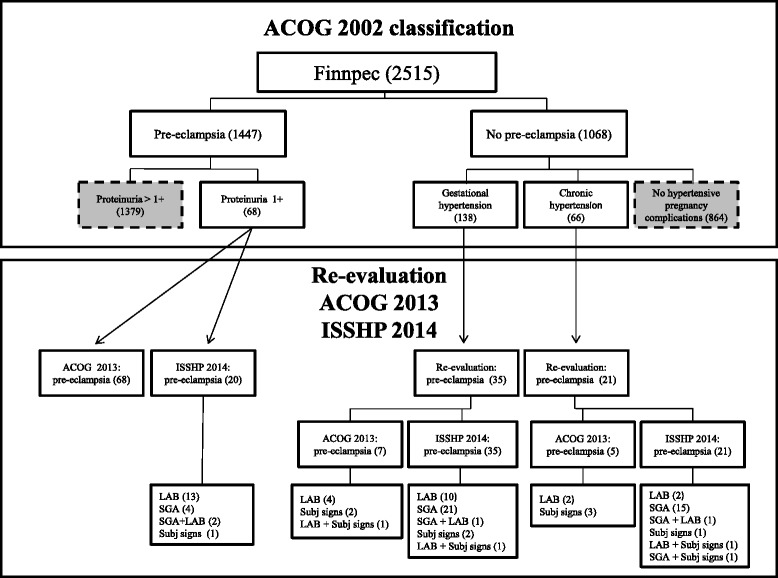
FINNPEC cohort December 16, 2015. The division of FINNPEC cohort into pre-eclamptic and non-pre-eclamptic women according to the ACOG 2002 classification and the new evaluation by the ACOG 2013 and ISSHP 2014 classifications. SGA = Small for gestational age, LAB = Laboratory findings, Subj signs = Subjective signs, PE = Pre-eclampsia

FINNPEC is a nationwide database of PE and non-PE women. At the time of writing this article on December 16, 2015, 2515 women were diagnosed (Fig. 1). Data was collected from maternity cards and hospital records from five university hospitals (Helsinki, Tampere, Kuopio, Oulu, Turku) during 2008–2011. Nulliparous or multiparous women with a singleton pregnancy were eligible for the study. Using the ACOG 2002 criteria, PE was defined as hypertension and proteinuria occurring after 20 weeks of gestation. Hypertension was defined as systolic blood pressure ≥140 mmHg or diastolic blood pressure ≥90 mmHg after 20 weeks of gestation. Proteinuria was defined as the urinary excretion of ≥0.3 g protein in a 24-h specimen, or 0.3 g/l, or two ≥1+ readings on dipstick in a random urine determination with no evidence of the urinary tract infection. Women who suffered from proteinuria without hypertension (*n* = 19) were included in the control group. Furthermore, women who suffered from gestational hypertension or chronic hypertension but did not meet the criteria for PE were included in the control group (*n* = 138 and *n* = 66 respectively).

Birth weights below -2.0 standard deviation (SD) units were classified as small-for-gestational age (SGA) according to Finnish standards (Pihkala 1989). Hemolysis, elevated liver enzymes, and low platelet count (HELLP) syndrome were diagnosed when at least two of the following criteria were met: lactate dehydrogenase **(**LD) ≥ 235 U/l, alanine aminotransferase (ALAT) ≥ 70 U/l, aspartate aminotransferase (ASAT) ≥ 70 U/l, thrombocytes ≤ 100 E9/l.

Each diagnosis was ascertained retrospectively based on hospital records and confirmed independently by a research nurse and a study physician. All subjects provided written informed consent, and the FINNPEC study protocol was approved by the coordinating Ethics Committee of the Hospital District of Helsinki and Uusimaa.

The summary of ACOG 2013 and ISSHP 2014 criteria is presented in Table 1. According to the two criteria, PE is defined as hypertension combined with proteinuria, or in absence of proteinuria, combined with at least one or more other findings including maternal organ dysfunction (elevated liver enzymes, haematological complications, renal insufficiency, neurological symptoms), pulmonary edema (ACOG 2013), and uteroplacental dysfunction (ISSHP 2014). Hypertension is classified either as new onset hypertension after 20 weeks of gestation with blood pressure levels ≥ 140/90 mmHg on two occasions at least 4 h apart, or as chronic hypertension. Some differences appear in laboratory measurements between the two criteria. Renal insufficiency is defined as creatinine levels > 100 μmol/l(1.1 mg/dL) and ≥ 90 μmol/l according to the ACOG 2013 and ISSHP 2014 criteria respectively. A low platelet count is defined as < 100 E^9/l and < 150 E^9/l according to the ACOG 2013 and ISSHP 2014 respectively. An impaired liver function is defined as elevated transaminases at least twice the upper limit of normal according to the two criteria. ACOG 2013 criteria include pulmonary oedema and cerebral or visual symptoms in subjective signs and present severe features of PE with any of the following findings: blood pressure ≥ 160/110 mmHg, thrombocytopenia < 100 E^9/l, at least doubled liver enzymes, severe persistent right upper quadrant pain or epigastric pain unresponsive to medication without any other reason or both, progressive renal insufficiency with at least doubled creatinine in the absence of any other renal disease, pulmonary edema, and new-onset cerebral and visual disturbances. ISSHP 2014 criteria include as a sign of liver involvement severe right upper quadrant or epigastric pain and as neurological complications e.g., eclampsia, altered mental status, blindness, stroke or more commonly hyperreflexia when accompanied by clonus, severe headaches when accompanied by hyperreflexia, and persistent visual scotomata. Haematological complications are defined as thrombocytopenia, hemolysis, and disseminated intravascular coagulation (DIC).

**Table 1:** Summary of the PE criteria according to the ACOG 2013 and the ISSHP 2014 classifications

Pre-eclampsia criteria	
ACOG 2013	ISSHP 2014
Blood pressure	
≥140 mmHg systolic and/or ≥90 mmHg diastolic	
Proteinuria	
≥300 mg/day urine protein/creatinine ≥30 mg/mmol	
≥1+ on dipstick testing	≥2+ on dipstick testing (>1 g/l)
Or in absence of proteinuria	
Liver transaminases > 2 × normal	
Platelet count	
PLT < 100 E9/l	PLT < 150 E9/l
Renal insufficiency	
Creatinine > 100 μmol/l	Creatinine ≥ 90 μmol/l
Subjective signs of PE	
	Uteroplacental dysfunction

*ACOG* The American College of Obstetricians and Gynecologists, *ISSHP* International Society for the Study of Hypertension in Pregnancy, *PLT* platelet count, *PE* pre-eclampsia

The most notable difference between the revised criteria of ACOG and ISSHP would be in regard to the definition of foetal growth restriction. ACOG defines foetal growth restriction as a sign of severe PE according to the 2002 criteria, but it was not included in the revised version, whereas according to the ISSHP criteria foetal growth restriction combined to hypertension meets the diagnostic criteria for PE. In the present study, we considered as subjective signs severe headache, apparent visual disturbances, epigastric pain, and hyperreflexia.

## Results

**Fig. 2:**
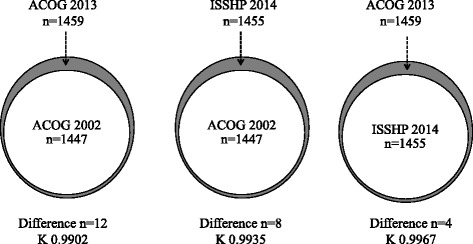
The number of women with pre-eclampsia according to the ACOG 2002 and 2013 and ISSHP 2014 classifications. Grey zone represents women diagnosed with PE only with one of the classifications but not with the other. K = Cohen’s kappa coefficient

The number of women with PE increased 0.8 % (1459/1447) according to the ACOG 2013 criteria and 0.6 % (1455/1447) according to the ISSHP 2014 criteria (Figs. 1 and 2). The summary of the findings in women with hypertension and proteinuria 1+ on dipstick and women with gestational or chronic hypertension diagnosed with PE according to the ACOG 2013 and the ISSHP 2014 classifications is presented in Tables 2, 3 and 4.

**Table 2:** Women with proteinuria +1 on dipstick diagnosed with pre-eclampsia according to the ISSHP 2014 criteria

	ISSHP 2014
Total	20
SGA	6
LAB	15
Subjective signs	1

Two of the women had both a SGA newborn and abnormal laboratory measurements

**Table 3:** Women with gestational hypertension diagnosed with pre-eclampsia according to the ACOG 2013 and ISSHP 2014 criteria

	ACOG 2013	ISSHP 2014
Total	7	35
SGA		22
LAB	5	12
Subjective signs	3	3

One of the women had abnormal laboratory findings in addition to subjective signs of PE according to ACOG and ISSHP criteria. One of the women had both a SGA newborn and abnormal laboratory measurements according to ISSHP

**Table 4:** Women with chronic hypertension diagnosed with pre-eclampsia according to the ACOG 2013 and the ISSHP 2014 criteria

	ACOG 2013	ISSHP 2014
Total	5	21
SGA		17
LAB	2	4
Subjective signs	3	3

According to the ISSHP 2014 criteria, one of the women had both a SGA newborn and abnormal laboratory findings, one woman both a SGA newborn and subjective signs, and one woman had abnormal laboratory findings and subjective signs

*SGA* small for gestational age, *LAB* laboratory findings

All 68 women with the amount of proteinuria not exceeding 1+ on dipstick diagnosed originally with PE met the ACOG 2013 criteria but only 20 women (29.4 %) met the ISSHP 2014 criteria. Of those 20 women diagnosed with PE according to the ISSHP 2014 criteria, 13 had abnormal laboratory measurements, four women had an infant with growth restriction, two women had abnormal laboratory measurements and an infant with growth restriction, and one woman had subjective signs. Nine women had a platelet count less than 150 E9/l but more than 100 E9/l, five women had a platelet count less than 100 E9/l, and eight women’s liver transaminases were at least twice the upper limit of normal. The woman with subjective symptoms suffered from visual disturbances, headaches, and vivid reflexes, as well as from twitches, nausea, and vomiting also post partum. She had no abnormal laboratory measurements and was treated with intravenous magnesium sulphate.

Of the 138 women with gestational hypertension according to the ACOG 2002 criteria, seven (5.1 %) and 35 (25.4 %) women were diagnosed with PE according to the ACOG 2013 and the ISSHP 2014 criteria respectively. Of those women who met the ACOG 2013 criteria, four women had abnormal laboratory measurements, two women had subjective signs, and one woman had both. Three women had a platelet count less than 100 E9/l and four women had liver transaminases twice the normal concentration. Of those 35 women who met the criteria of ISSHP 2014, 10 women had abnormal laboratory measurements, 21 had an infant with growth restriction, and one woman had both. Two women had subjective signs and one woman had abnormal laboratory measurements and subjective signs. As for the abnormal laboratory measurements, eight women had a platelet count less than 150 E9/l but more than 100 E9/l, three women had a platelet count less than 100 E9/l, and four women had liver transaminases at least twice the upper limit of normal. All women with subjective signs were treated with intravenous magnesium sulphate. Subjective signs included epigastric pain, intense headache, visual disturbances, and vivid reflexes. Sixty-three percent of women with gestational hypertension who were diagnosed with PE according to the ISSHP criteria had an infant with growth restriction. In addition, abnormal laboratory findings were found more than two times more often according to the ISSHP 2014 criteria (12) than according to the ACOG 2013 criteria (5). This was mainly due to low platelet counts, and six of the women were diagnosed with PE only because of platelet counts less than 150 E9/l.

Five (7.6 %) and 21 (31.8 %) of the 66 women with chronic hypertension were diagnosed with PE according to the ACOG 2013 and the ISSHP 2014 criteria, respectively. Of those women who met the ACOG 2013 criteria, 2 women had abnormal laboratory findings and 3 women had subjective signs. Laboratory findings for both women included a platelet count less than 150 E9/l but more than 100 E9/l and liver transaminases at least twice the upper limit of normal. Of those 21 women who met the ISSHP 2014 criteria, 2 women had abnormal laboratory findings, 15 women had an infant with growth restriction, one woman had both abnormal laboratory findings and an infant with growth restriction. One woman suffered from subjective signs, one woman had in addition to subjective signs also abnormal laboratory findings, and one woman in addition to subjective signs an infant with growth restriction. Subjective signs of the three women included hyperreflexia, headaches, epigastric pain, nausea, vomiting and visual disturbances. Eighty-one percent of the women with chronic hypertension and diagnosed with PE according to the ISSHP criteria had an infant with growth restriction.

Four of the seven women with gestational hypertension diagnosed with PE according to the ACOG 2013 criteria had antihypertensive medication, six deliveries were induced because of the hypertension or other symptoms anticipating PE, and one woman underwent a caesarean section. Two of these women gave birth before 37 + 0 weeks of gestation. Nineteen of the 35 women with gestational hypertension diagnosed with PE according to the ISSHP 2014 criteria had antihypertensive medication, 10 deliveries were induced, and 20 women underwent a caesarean section. Six women gave birth before 34 + 0 weeks of gestation and eight before 37 + 0 weeks of gestation.

All of the five women with chronic hypertension diagnosed with PE according to the ACOG 2013 criteria had antihypertensive medication. Two deliveries were induced because of the hypertension or other symptoms anticipating PE, and two women underwent caesarean section. Three women gave birth before 37 + 0 weeks of gestation. Seventeen of the 21 women with chronic hypertension diagnosed with PE according to the ISSHP 2014 criteria had antihypertensive medication, four deliveries were induced, and 14 women underwent caesarean section. Twelve women gave birth before 34 + 0 weeks of gestation.

## Discussion

In this study, we examined how the ACOG 2013 and the ISSHP 2014 PE classifications affected previously ascertained PE diagnoses in the FINNPEC cohort defined according to the ACOG 2002 classification. Our results showed only minor changes in the total number of affected women. There were noticeable changes within three subgroups. All PE women with proteinuria not exceeding 1+ on dipstick remained PE according to the ACOG 2013 criteria but less than one-third according to the ISSHP 2014 criteria. More than one in four of the women with gestational and chronic hypertension were diagnosed with PE according to the revised criteria, when proteinuria was considered optional in the presence of other findings. A challenge to the validity of the study design is the selection of the control group (non-PE women). Women with some symptoms and signs of PE, which were, however, not enough to satisfy the diagnostic criteria, are overrepresented in the control group.

There is a need to better understand the pathophysiology of PE and to define short- and long-term prognoses. The revised classifications by the ACOG and the ISSHP, which change the paradigm that the diagnosis of PE always requires proteinuria, provide broader definitions of the disease, which seems justified in a heterogeneous disease with diverse clinical presentations**.** To the best of our knowledge, this is the first study to re-evaluate PE diagnosis according to the new classifications in a cohort of PE and non-PE women. The strength of the study is a carefully characterised cohort with comprehensive clinical and background information from each study subject verified from maternity cards and hospital records. Blood pressure and urine measurements are performed regularly during the course of the pregnancy in maternity clinics and documented in maternity cards. The weakness of the study is the retrospective design, which may introduce biases. Ideally the three definitions should to be applied and compared in a population of pregnant women without any previous selection or filter. Given the incidence of PE in the general population, to get significant results it would need a prospective study design with a large number of unselected pregnant women.

One challenge in applying retrospectively the revised criteria is inconsistency in recording subjective signs and symptoms, the importance of which is increased in the diagnostic criteria of PE especially when proteinuria is absent. Only few women had signs and symptoms that were severe enough to be considered PE related when proteinuria was absent or not exceeding 1+ on dipstick. Many women had signs and symptoms weaker than the ones defined in the revised criteria. The symptoms of these women included transient visual disturbances, headaches not always responding to medication but eventually ending spontaneously, transient epigastric sensations and pain, and vivid reflexes, albeit without noteworthy clonus. The amount of available laboratory measurements varies. For example, in cases where PE is suspected late in pregnancy and delivery is warranted, laboratory measurements might be scarce or not available. The diagnosis of PE might remain unconfirmed if not symptomatic postpartum. Moreover, protocols of laboratory measurements when PE is suspected are not harmonised between the hospitals. This might affect how care providers react to PE symptoms when proteinuria is absent. To minimise the bias, only one abnormal measurement was not considered significant enough in the present study.

In Finnish maternity care, all women with hypertension in pregnancy are carefully monitored in case of developing proteinuria, foetal growth restriction, or new-onset PE. Treatment of PE is still limited to antihypertensive medication, eclampsia prophylaxis, and indicated delivery. Identifying patients with a more broad definition of PE does not necessarily have a major effect to the course of the disease. When signs and symptoms suggested PE, women are treated as if they were diagnosed with PE even if they did not fulfil all diagnostic criteria. This was also seen in the FINNPEC cohort. Women were already treated as PE patients when diagnosed with gestational hypertension. In some other countries where management is more conservative for pregnancy related hypertension, the findings may be more relevant. Moreover, broader definitions might provide better opportunities for earlier recognition of PE and warrant closer observation. A more sensitive recognition of PE might also be useful in the prevention of non-communicable diseases later in life. Women with early onset (requiring delivery before 34 weeks of gestation), recurrent, or preterm PE are at the highest risk for cardiovascular diseases [4, 10]. However, the risk is still noticeable among all PE women.

According to the ISSHP 2014 classification the women included in the PE group were predominantly the ones with foetal growth restriction. They are likely to have had more adverse maternal and neonatal outcomes. On the other hand, the ones excluded from the PE group were those with minimal proteinuria and milder disease. Thus, patients diagnosed with PE might have more severe form of the disease. It would be interesting to see in the future studies how maternal and neonatal outcomes change for the group of PE patients classified by the ISSHP 2014 criteria.

For purposes of clinical practice and research, demands for diagnostic criteria of PE are different. More rigid criteria are usually applied in research, whereas in clinical practice sensitivity is more important than specificity. Until more specific criteria are available for PE, diagnosis relies on clinical classifications. Biomarkers and genetic variants are candidates which are expected to allow the refining of diagnostic and prognostic subgroups. Furthermore, they may clarify the role of known risk factors, e.g., obesity and diabetes in the disease pathogenesis. Defining better diagnostic criteria is a continuous challenge in this heterogeneous disorder. In future, prospective studies comparing the different criteria in a larger cohort may be appropriate to ascertain any significant difference regarding the time of diagnosis as a potential benefit in terms of changing managements as well as evaluating maternal and neonatal outcomes.

## Conclusion

Based on our results, the two revised classifications have no remarkable effect on the total number of women diagnosed with PE in the FINNPEC cohort. The main difference between ACOG 2013 and ISSHP 2014 criteria would be in regard to the definition of foetal growth restriction and the amount of proteinuria. The revised classifications of the ACOG 2013 and the ISSHP 2014 enable more sensitive diagnostics of PE in women with new-onset signs and symptoms of PE when proteinuria is absent. The new classifications reflect the heterogeneity of PE.

## Abbreviations

ACOG, The American College of Obstetricians and Gynecologists; ISSHP, International Society for the Study of Hypertension in Pregnancy; LAB, laboratory findings; PE, pre-eclampsia; PLT, platelet count; SGA, small for gestational age; Subj signs, subjective signs; K, Cohen’s kappa coefficient
